# Disparities in cause-specific mortality by health insurance type and premium: evidence from Korean NHIS-HEALS cohort study, 2002–2019

**DOI:** 10.1186/s12889-024-19088-3

**Published:** 2024-06-12

**Authors:** Ye-Seul Kim, Joungyoun Kim, Yonghoon Kim, Hee-Taik Kang

**Affiliations:** 1 Department of Family Medicine, Chungbuk National University Hospital, 776 1-Soonwhan-Ro, Seowon-Gu, Cheongju, 28644 Republic of Korea; 2 Department of Artificial Intelligence, University of Seoul, 163 Seoulsiripdae-Ro, Dongdaemun-Gu, Seoul, 02504 Republic of Korea; 3 Department of Biostatistics and Computing, Yonsei University Graduate School, 50-1 Yonsei-Ro, Seodaemun-Gu, Seoul, 03722 Republic of Korea; 4 Department of Family Medicine, Yonsei University College of Medicine, 50-1 Yonsei-Ro, Seodaemun-Gu, Seoul, 03722 Republic of Korea; 5 Institute for Innovation in Digital Healthcare, Yonsei University, 50-1 Yonsei-Ro, Seodaemun-Gu, Seoul, 03722 Republic of Korea

**Keywords:** Health status disparity, Socioeconomic factors, Health insurance, Mortality, Cause of death, Health programs

## Abstract

**Background:**

Although one’s socioeconomic status affects health outcomes, limited research explored how South Korea’s National Health Insurance (NHI) system affects mortality rates. This study investigated whether health insurance type and insurance premiums are associated with mortality.

**Methods:**

Based on the National Health Insurance Service-Health Screening cohort, 246,172 men and 206,534 women aged ≥ 40 years at baseline (2002–2003) were included and followed until 2019. Health insurance type was categorized as employee-insured (EI) or self-employed-insured (SI). To define low, medium, and high economic status groups, we used insurance premiums at baseline. Death was determined using the date and cause of death included in the cohort. Cox proportional hazard models were used to analyze the association between insurance factors and the overall and cause-specific mortality.

**Results:**

The SI group had a significantly higher risk of overall death compared to the EI group (adjusted hazard ratio (HR) [95% confidence interval]: 1.13 [1.10–1.15] for men and 1.18 [1.15–1.22] for women), after adjusting for various factors. This trend extended to death from the five major causes of death in South Korea (cancer, cardiovascular disease, cerebrovascular disease, pneumonia, and intentional self-harm) and from external causes, with a higher risk of death in the SI group (vs. the EI group). Further analysis stratified by economic status revealed that individuals with lower economic status faced higher risk of overall death and cause-specific mortality in both sexes, compared to those with high economic status for both health insurance types.

**Conclusion:**

This nationwide study found that the SI group and those with lower economic status faced higher risk of overall mortality and death from the five major causes in South Korea. These findings highlight the potential disparities in health outcomes within the NHI system. To address these gaps, strategies should target risk factors for death at the individual level and governments should incorporate such strategies into public health policy development at the population level.

**Trial registration:**

This study was approved by the Institutional Review Board of Chungbuk National University Hospital (CBNUH-202211-HR-0236) and adhered to the principles of the Declaration of Helsinki (1975).

**Supplementary Information:**

The online version contains supplementary material available at 10.1186/s12889-024-19088-3.

## Background

Mortality rates are a key indicator of population health and are influenced by a complex interplay of multiple factors. These factors encompass both unmodifiable determinants, such as sex, age, and race/ethnicity, and modifiable factors, such as lifestyle choices (smoking, diet, and physical activity), occupation, education, and socioeconomic status (SES) [1, 2]. As a result of advancement in medicine and research, the importance of prevention and education alongside disease treatment for reducing mortality risk is gaining recognition. Policymakers are also increasingly focusing on addressing health outcome disparities arising from individual and national differences [3].

SES is a broad concept that encompasses factors such as education, income, and occupation and significantly impacts health outcome. Numerous studies have established a link between SES and health disparities, thus influencing medical utilization, health behaviors, and ultimately mortality [2]. The theory that these social and environmental factors influence health outcomes is known as the “social determinants of health” [4]. For instance, previous research has shown that individuals with lower educational levels experience higher mortality rates from various causes of death, and a wider life expectancy gap is associated with bigger educational disparities [5, 6]. Income level has also been identified as a key factor in health disparities that influences medical utilization, health behavior, and life expectancy [7–10]. Additionally, health insurance coverage and premium levels can contribute to health disparities [11, 12]. For example, Pulte et al. found lower survival rates for patients without insurance or Medicaid cover than for those with other forms of insurance [13]. Similarly, population that is at risk of lacking stable health insurance coverage, including racial and ethnic minorities and those with lower income, is likely to have worse health status [14]. These findings highlight the influence of SES and health insurance status on health disparities and their associated outcomes.

While prior international research links SES, health insurance, and mortality, the impact within South Korea’s unique public health insurance system remains unclear. This study addresses this gap by investigating the association between mortality risk and the health insurance type (employee insured [EI] vs. self-employed insured [SI]) and insurance premium level. We hypothesize that individuals with lower economic status and employment insecurity will be associated with higher risk of death compared to those with higher economic status and secure employment status in South Korea. We investigate the differences in overall and cause-specific mortality risks based on health insurance type and insurance premium using the Korean National Health Insurance Service-Health Screening (NHIS-HEALS) cohort.

## Material and methods

### South Korea’s NHIS and NHIS-HEALS cohort

The NHIS is a single-payer, mandatory health insurance program that covers all citizens who reside in South Korea, except Medical Aid beneficiaries. The NHIS divides its enrollees into two main categories: the EI and dependents (70%) and the SI and dependents (27%) [15]. The EI covers workers and employers in all workplaces, including public officials, private school employees, and daily paid workers at construction sites. The SI covers those not in the EI group and their dependents, including farmers, fishers, and self-employed persons. The NHIS enrollees are mandated to pay premiums based on their income or assets. In the EI group, insurance premiums are charged based on employees’ monthly average wages, and in the SI group, they are charged based on information on the household’s wealth, such as income, property, and cars owned [16]. The NHIS uses collected premiums to cover a portion of subscribers' medical expenses through co-payments. The NHIS uses collected premiums to cover a portion of subscribers’ medical expenses through co-payments. The NHI offers a wide range of medical services, including inpatient care, outpatient clinic visits, prescription drugs, and preventive healthcare services to their enrollees.

Leveraging data from the NHIS, the NHIS-HEALS cohort database includes healthcare usage, death-related information, and health-screening information. The variables from the NHIS were demographics, date of death, cause of death, income-based insurance premiums (a proxy for household income), prescription records, and diagnostic codes based on the 10th edition of the International Classification of Diseases (ICD-10). Blood pressure, anthropometric measurements, laboratory results, answers to a self-questionnaire about lifestyle (smoking status, alcohol consumption, and physical activity), and personal and family medical histories were obtained [17].

This study analyzed the NHIS-HEALS cohort database from 2002 to 2019. This database comprised a 10% sample of adults aged 40–79 years who underwent national health screening programs (NHSPs) and were enrolled in the NHIS program in 2002 and 2003.

The ethics committee of the NHIS waived the need for informed consent because data from the NHIS-HEALS were anonymized at all stages and de-identified, including through data cleaning and statistical analysis. This study was approved by the Institutional Review Board of Chungbuk National University Hospital (CBNUH-202211-HR-0236) and adhered to the principles of the Declaration of Helsinki (1975).

### Study population and definition of study group

**Fig. 1:**
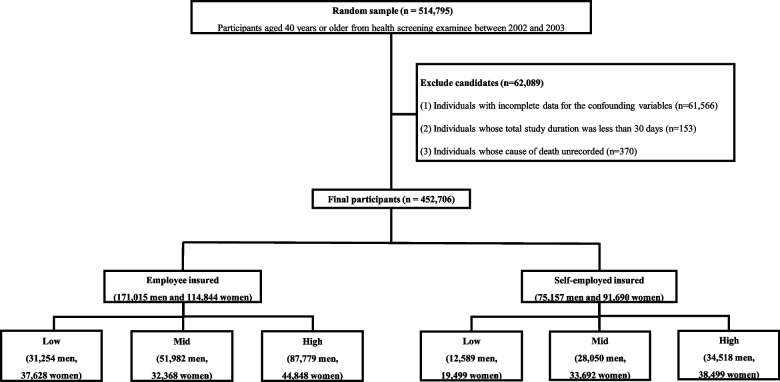
Flow chart of inclusion and exclusion criteria of participants

Participants aged 40 years or older who underwent NHSPs in 2002 and 2003 were included in the NHIS-HEALS cohort database. Of the initial 514,795 participants, 62,089 were excluded based on the following exclusion criteria: 1) individuals with incomplete data for the confounding variables (*n* = 61,566); 2) individuals whose total study duration was less than 30 days (*n* = 153); and 3) individuals without a recorded cause of death (*n* = 370). After exclusion, 452,706 individuals (246,172 men and 206,534 women) were included in the study. A flowchart of the inclusion and exclusion criteria of this study is shown in Fig. 1.

The study group was classified based on health insurance type and insurance premiums in the NHIS-HEALS cohort database. Health insurance was divided into the EI and SI groups. Insurance premiums were used to define economic status group:1–3rd deciles as low, 4–7th deciles as mid, and 8–10th deciles as high. We categorized the study group into a total of six groups according to the combination of these two factors: low, mid, and high for EI, and low, mid, and high for SI.

Following this classification, 285,859 individuals (171,015 men and 114,844 women) were in the EI group, and 166,847 individuals (75,157 men and 91,690 women) were in the SI group.

### Outcomes

The primary purpose of this study was to compare the risk of overall death by health insurance type and economic status. Overall death was defined as all death after enrollment (2002–2003). Death was defined using death information from the NHIS-HEALS cohort database (date of death and direct cause of death).

As a secondary outcome, the risk of death due to each cause, including the five major causes of death in South Korea and external causes, was analyzed [18]. The causes of death were classified by the death certificate and ICD-10: (1) cancer (ICD-10 codes C00–D48); (2) cardiovascular disease (CVD; I20–I51); (3) cerebrovascular disease (CbVD; I60–I69); (4) pneumonia (J12–J18); (5) intentional self-harm (X60–X84); and (6) external cause (V01–V99, W00–W19, W20–W99, X00–X59, and Y00–Y8).

### Duration of study

The study duration was defined as the period between a participant’s national health examination and their date of death. The start date of the study was defined as the day of the first health examination conducted between 2002 and 2003. For participants who died after enrollment and before December 31, 2019, the study ended on their date of death. For participants who remained alive until December 31, 2019, the study ended on the later of the dates for their last outpatient clinic visit, last health screening, or the last day the participants took the prescribed medication.

### Covariates

We included potential death risk factors from the NHIS-HEALS cohort data as covariates in our analysis. Body mass index (BMI, kg/m^2^) was calculated as body weight (kg) divided by height squared (m^2^). Smoking status, alcohol consumption, physical activity status, and medical history were collected via self-reported questionnaires from the NHSPs and classified as follows: Smoking status was categorized as never, former, or current smokers. Never smokers were defined as individuals who answered “Never” to “Do you smoke?” Former smokers were defined as individuals who responded “No. I smoked earlier, but not currently” to this question. Current smokers were defined as individuals who answered “Yes, I currently smoke cigarettes.” Alcohol consumption was classified as rare (one drink per month or less), moderate (two drinks per month or more to two drinks per week), or heavy (three drinks or more per week). Physical activity was divided into three categories based on the answer to “How many times a week do you exercise enough to make you sweat?”: rare (less than once per week), sometimes (one to four days per week), and regular (five or more days per week) [19].

Residential areas were divided into the Seoul capital area (including Seoul, Gyeonggi-do, and Incheon metropolitan city), other metropolitan cities (including Busan, Daegu, Daejeon, Kwangju, and Ulsan, where the population is 500,000 or more in large cities other than the Seoul capital area), and non-metropolitan areas.

Charlson’s comorbidity index (CCI) was used to categorize patient comorbidities based on the ICD codes recorded in the administrative system [20]. The CCI scores correlated well with patient death or medical resource utilization [21]. Individuals with higher CCI scores were more likely to die or use medical resources. Each comorbidity had weighted scores of 1 to 6 based on the adjusted risk of death or medical resource utilization [22]. The CCI score was calculated using the ICD-10 code entered within 1 year of enrollment, and the sum of CCI scores was recategorized into four groups (0, 1, 2, and 3 or more).

Medical history of cancer, CVD, and CbVD was classified as having a history when the subject indicated it in the self-questionnaire at the time of study enrollment or when registration of the main diagnosis was confirmed within one year (cancer, C00–D48; CVD, I20–I25; and CbVD, I60–I69).

### Statistical analyses

Continuous variables (age, BMI, systolic blood pressure, fasting glucose, total cholesterol, and alanine aminotransferase [ALT]) were presented as the mean (95% confidence interval). Categorical variables (cigarette smoking, alcohol consumption, physical activity, residential area, medical history of cancer, CVD, CbVD, and CCI) were expressed as the number of participants (percentage). To compare the mean or percentage of each variable, analysis of variance for continuous variables and the chi-square test for categorical variables were performed. Kaplan–Meier estimates and the log-rank test were conducted to compare mortality rates among the study groups to ascertain whether insurance type and insurance premium affected survival rates.

Cox proportional hazards regression models were constructed to investigate the association between mortality risk and the insurance type and premium, after controlling the following variables: 1) Model 1, age only; 2) Model 2, smoking status (never, former, and current), alcohol consumption (rare, moderate, and heavy), physical activity (rare, sometimes, and regular), and residential area (Seoul capital, other metropolitan, and non-metropolitan), added to Model 1; and 3) Model 3, BMI, systolic blood pressure, fasting glucose, ALT, total cholesterol, and CCI (0, 1, 2, and ≥ 3), added to Model 2. In addition, Cox proportional hazards regression models were examined after stratification into each subgroup with the history of cancer, CVD, and CbVD. The Cochran-Armitage test was conducted to analyze the linear trend between insurance premiums and mortality risk, and the results were described as P-trends.

Statistical analyses were conducted from September 1, 2023, to April 22, 2024, using the statistical packages SAS Enterprise version 7.1 (SAS Inc., Cary, NC, USA) and R studio version 3.3.3 (The R Foundation, Vienna, Austria). All *p*-values were two-sided, and statistical significance was set at *p* < 0.05.

## Results

### Baseline characteristics of participants

Table 1 shows the baseline characteristics of the participants according to health insurance type and economic status. Compared to the EI group, the SI group had a higher prevalence of heavy drinkers and individuals with a history of cancer and CVD in both sexes; women in the SI group had a higher BMI, proportion of current smokers, and individuals with a history of CbVD. Within the same health insurance type group, participants with lower economic status exhibited higher rates of current smoking, heavy alcohol consumption, and physical inactivity. This observation suggests that individuals with lower economic status tended to have less healthy lifestyle habits. SI group members with a lower economic status had a higher risk of having a CCI score of ≥ 3 and a higher prevalence of having cancer, CVD, and CbVD history.

**Table 1:** Baseline characteristics of study participants according to insurance type and economic status (2002–2003)

	**Employee insured**					**Self-employed insured**				
	**Total**	**High**	**Mid**	**Low**	***p*-value**	**Total**	**High**	**Mid**	**Low**	***p*-value**
**a) Men**										
Number	171,015	87,779	51,982	31,254		75,157	34,518	28,050	12,589	
Age, years	50.9 (42.0–59.8)	49.7 (40.8–58.6)	51 (42.4–59.6)	54.2 (45.8–62.6)	< 0.001	54.7 (44.8–64.6)	53.8 (44.8–62.8)	54.0 (44.1–63.9)	58.8 (47.6–70)	< 0.001
Body mass index, kg/m^2^	24.0 (21.2–26.8)	24.1 (21.4–26.8)	23.8 (20.9–26.7)	23.9 (20.9–26.9)	< 0.001	24.0 (21.0–27.0)	24.4 (21.6–27.2)	23.9 (20.9–26.9)	23.4 (20.3–26.5)	< 0.001
Systolic blood pressure, mmHg	128.7 (111.6–145.8)	126.9 (110.6–143.2)	129.6 (112.2–147.0)	131.9 (113.7–150.1)	< 0.001	128.9 (110.6–147.2)	128.0 (110.3–145.7)	129.0 (110.5–147.5)	131.2 (112.1–150.3)	< 0.001
Hemoglobin, g/dL	14.9 (13.8–16.0)	14.9 (13.8–16.0)	14.8 (13.7–15.9)	14.7 (13.6–15.8)	< 0.001	14.8 (13.6–16.0)	14.9 (13.7–16.1)	14.7 (13.5–15.9)	14.6 (13.3–15.9)	< 0.001
Fasting glucose, mg/dL	99.7 (64.4–135.0)	97.9 (67.0–128.8)	100.4 (64.3–136.5)	103.8 (59.9–147.7)	< 0.001	101.3 (63.8–138.8)	101.4 (65.4–137.4)	101.0 (62.5–139.5)	101.9 (62.7–141.1)	0.068
ALT, mg/dL	30.1 (6.3–53.9)	30.2 (7.4–53.0)	30.1 (4.6–55.6)	29.8 (6.5–53.1)	< 0.001	30.2 (6.1–54.3)	30.0 (7.2–52.8)	30.7 (6.2–55.2)	29.6 (3.1–56.1)	< 0.001
Total cholesterol, mg/dL	199.3 (161.7–236.9)	200.2 (163.4–237.0)	197.4 (159.8–235.0)	199.9 (160.2–239.6)	< 0.001	197.7 (158.6–236.8)	199.9 (161.2–238.6)	196.5 (157.3–235.7)	194.2 (154.5–233.9)	< 0.001
Smoking status, N (%)					< 0.001					< 0.001
Never-smokers	69,152 (40.4)	36,269 (41.3)	20,350 (39.1)	12,533 (40.1)		32,893 (43.8)	16,277 (47.2)	11,538 (41.1)	5078 (40.3)	
Former smokers	27,282 (16.0)	16,471 (18.8)	7117 (13.7)	3694 (11.8)		11,030 (14.7)	5533 (16.0)	3891 (13.9)	1606 (12.8)	
Current smokers	74,581 (43.6)	35,039 (39.9)	24,515 (47.2)	15,027 (48.1)		31,234 (41.6)	12,708 (36.8)	12,621 (45.0)	5905 (46.9)	
Alcohol consumption, N (%)					< 0.001					< 0.001
Rare	56,177 (32.8)	26,779 (30.5)	17,609 (33.9)	11,789 (37.7)		29,278 (39.0)	12,924 (37.4)	10,988 (39.2)	5366 (42.7)	
Moderate	86,645 (50.7)	48,931 (55.7)	24,244 (46.6)	13,470 (43.1)		26,680 (35.5)	13,266 (38.4)	9424 (33.6)	3990 (31.7)	
Heavy	28,193 (16.5)	12,069 (13.8)	10,129 (19.5)	5995 (19.2)		19,199 (25.5)	8328 (24.1)	7638 (27.2)	3233 (25.7)	
Physical activity, N (%)					< 0.001					< 0.001
Rare	76,802 (44.9)	34,558 (39.4)	26,163 (50.3)	16,081 (51.5)		44,298 (58.9)	17,948 (52.0)	18,040 (64.3)	8310 (66.0)	
Sometimes	78,812 (46.1)	45,134 (51.4)	21,243 (40.9)	12,435 (39.8)		22,331 (29.7)	12,189 (35.3)	7200 (25.7)	2922 (23.2)	
Regular	15,401 (9.0)	8087 (9.2)	4576 (8.8)	2738 (8.8)		8548 (11.4)	4381 (12.7)	2810 (10.0)	1357 (10.8)	
Residential area, N (%)					< 0.001					< 0.001
Seoul capital area	75,439 (44.1)	37,842 (43.1)	23,713 (45.6)	13,884 (44.4)		27,383 (36.4)	15,149 (43.9)	8619 (30.7)	3615 (28.7)	
Other metropolitan cities	43,867 (25.7)	22,752 (25.9)	12,673 (24.4)	8442 (27.0)		17,503 (23.3)	9221 (26.7)	6082 (21.7)	2200 (17.5)	
Non-metropolitan area	51,709 (30.2)	27,185 (31.0)	15,596 (30.0)	8928 (28.6)		30,271 (40.3)	10,148 (29.4)	13,349 (47.6)	6774 (53.8)	
CCI, N (%)					< 0.001					< 0.001
0	115,732 (67.7)	59,930 (68.3)	35,112 (67.5)	20,690 (66.2)		44,182 (58.8)	20,387 (59.1)	16,784 (59.8)	7011 (55.7)	
1	35,030 (20.5)	17,707 (20.2)	10,705 (20.6)	6,618 (21.2)		18,009 (24.0)	8319 (24.1)	6698 (23.9)	2992 (23.8)	
2	11,630 (6.8)	5772 (6.6)	3634 (7.0)	2224 (7.1)		7050 (9.4)	3230 (9.4)	2501 (8.9)	1319 (10.5)	
≥ 3	8623 (5.0)	4370 (5.0)	2531 (4.9)	1722 (5.5)		5916 (7.9)	2582 (7.5)	2067 (7.4)	1267 (10.1)	
History of cancer, N (%)	7594 (4.4)	4170 (4.8)	2114 (4.1)	1310 (4.2)	< 0.001	4199 (5.6)	2036 (5.9)	1421 (5.1)	742 (5.9)	< 0.001
History of CVD, N (%)	6134 (3.6)	3228 (3.7)	1763 (3.4)	1143 (3.7)	0.016	3656 (4.9)	1761 (5.1)	1236 (4.4)	659 (5.3)	< 0.001
History of CbVD, N (%)	2790 (1.6)	1374 (1.6)	842 (1.6)	574 (1.8)	0.005	2126 (2.8)	920 (2.7)	719 (2.6)	487 (3.9)	< 0.001
**b) Women**										
Number	114,844	44,848	32,368	37,628		91,690	38,499	33,692	19,499	
Age, years	53.0 (43.5–62.5)	54.3 (43.5–65.1)	53.8 (44.9–62.7)	50.9 (43.0–58.8)	< 0.001	54.5 (44.4–64.6)	52.6 (43.6–61.6)	53.8 (44.0–63.6)	59.4 (48.5–70.3)	< 0.001
Body mass index, kg/m^2^	23.9 (20.3–27.5)	23.6 (20.6–26.6)	24.1 (21.0–27.2)	24 (21.0–27.0)	< 0.001	24.2 (21.0–27.4)	24.2 (21.1–27.3)	24.2 (21.0–27.4)	24.1 (20.7–27.5)	< 0.001
Systolic blood pressure, mmHg	124.8 (106.4–143.2)	123.7 (105.2–142.2)	125.5 (107.0–144.0)	125.4 (107.4–143.4)	< 0.001	125.5 (106.1–144.9)	123.6 (105.0–142.2)	125.6 (106.2–145.0)	129.2 (108.6–149.8)	0.001
Hemoglobin, g/dL	12.9 (11.7–14.1)	12.8 (11.6–14.0)	12.9 (11.7–14.1)	12.8 (11.6–14.0)	< 0.001	12.9 (11.7–14.1)	12.9 (11.7–14.1)	12.9 (11.7–14.1)	12.9 (11.7–14.1)	< 0.001
Fasting glucose, mg/dL	95.3 (64.5–126.1)	95.0 (64.6–125.4)	96.1 (65.6–126.6)	95.0 (63.4–126.6)	< 0.001	96.6 (62.6–130.6)	95.4 (62.9–127.9)	96.7 (62.8–130.6)	98.7 (61.8–135.6)	< 0.001
ALT, mg/dL	21.0 (4.9–37.1)	20.6 (5.3–35.9)	21.5 (4.2–38.8)	20.9 (5.1–36.7)	< 0.001	21.7 (2.9–40.5)	21.5 (2.3–40.7)	21.8 (3.1–40.5)	22.1 (4.2–40.0)	0.001
Total cholesterol, mg/dL	202.2 (163.1–241.3)	202.3 (163.4–241.2)	203.5 (164.1–242.9)	200.9 (161.8–240.0)	< 0.001	203.1 (163.5–242.7)	202.4 (163.2–241.6)	202.3 (162.8–241.8)	205.8 (165.2–246.4)	< 0.001
Smoking status, N (%)					< 0.001					< 0.001
Never-smokers	111,787 (97.3)	43,705 (97.4)	31,415 (97.0)	36,667 (97.4)		86,673 (94.5)	37,213 (96.7)	31,716 (94.1)	17,744 (91.0)	
Former smokers	969 (0.8)	410 (0.9)	270 (0.8)	289 (0.8)		1005 (1.1)	350 (0.9)	397 (1.2)	258 (1.3)	
Current smokers	2088 (1.8)	733 (1.6)	683 (2.1)	672 (1.8)		4012 (4.4)	936 (2.4)	1579 (4.7)	1497 (7.7)	
Alcohol consumption, N (%)					< 0.001					< 0.001
Rare	95,298 (83.0)	38,649 (86.1)	26,843 (82.9)	29,806 (79.2)		75,014 (81.8)	31,610 (82.1)	27,438 (81.4)	15,966 (81.9)	
Moderate	18,059 (15.7)	5758 (12.8)	5039 (15.6)	7262 (19.3)		14,448 (15.8)	6157 (16.0)	5365 (15.9)	2926 (15.0)	
Heavy	1487 (1.3)	441 (1.0)	486 (1.5)	560 (1.5)		2228 (2.4)	732 (1.9)	889 (2.6)	607 (3.1)	
Physical activity, N (%)					< 0.001					< 0.001
Rare	74,313 (64.7)	25,822 (57.5)	21,723 (67.1)	26,768 (71.1)		63,025 (68.7)	23,749 (61.7)	24,319 (72.2)	14,957 (76.7)	
Sometimes	30,638 (26.7)	14,549 (32.4)	7840 (24.2)	8249 (21.9)		19,709 (21.5)	10,399 (27.0)	6351 (18.9)	2959 (15.2)	
Regular	9893 (8.6)	4477 (10.0)	2805 (8.7)	2611 (6.9)		8956 (9.8)	4351 (11.3)	3022 (9.0)	1583 (8.1)	
Residential area, N (%)					< 0.001					< 0.001
Seoul capital area	46,595 (40.6)	17,595 (39.2)	13,162 (40.7)	15,838 (42.1)		32,860 (35.8)	16,928 (44.0)	10,701 (31.8)	5231 (26.8)	
Other metropolitan cities	28,826 (25.1)	11,616 (25.9)	7677 (23.7)	9533 (25.3)		21,963 (24.0)	10,453 (27.1)	7802 (23.2)	3708 (19.0)	
Non-metropolitan area	39,423 (34.3)	15,637 (34.9)	11,529 (35.6)	12,257 (32.6)		36,867 (40.2)	11,118 (28.9)	15,189 (45.1)	10,560 (54.2)	
CCI, N (%)					< 0.001					< 0.001
0	65,230 (56.8)	24,746 (55.1)	17,619 (54.4)	22,865 (60.8)		49,105 (53.6)	21,311 (55.4)	18,251 (54.2)	9543 (48.9)	
1	30,364 (26.4)	11,846 (26.4)	8855 (27.3)	9663 (25.7)		25,101 (27.4)	10,429 (27.1)	9238 (27.4)	5434 (27.9)	
2	11,862 (10.3)	4825 (10.8)	3644 (11.3)	3393 (9.0)		10,362 (11.3)	4056 (10.5)	3729 (11.1)	2577 (13.2)	
≥ 3	7388 (6.4)	3431 (7.7)	2250 (7.0)	1707 (4.5)		7122 (7.8)	2703 (7.0)	2474 (7.3)	1945 (10.0)	
History of cancer, N (%)	5649 (4.9)	2542 (5.7)	1636 (5.1)	1471 (3.9)	< 0.001	4708 (5.1)	2132 (5.5)	1608 (4.8)	968 (5.0)	< 0.001
History of CVD, N (%)	5105 (4.4)	2391 (5.3)	1549 (4.8)	1165 (3.1)	< 0.001	4676 (5.1)	1868 (4.9)	1584 (4.7)	1224 (6.3)	< 0.001
History of CbVD, N (%)	2422 (2.1)	1156 (2.6)	759 (2.3)	507 (1.3)	< 0.001	2288 (2.5)	865 (2.2)	745 (2.2)	678 (3.5)	< 0.001

Values are presented as n (%) or mean (95% confidence interval)

*ALT* Alanine aminotransferase, *CCI* Charlson comorbidity index, *CVD* Cardiovascular disease, and *CbVD* Cerebrovascular disease

### Overall and cause-specific mortality by health insurance type

**Fig. 2:**
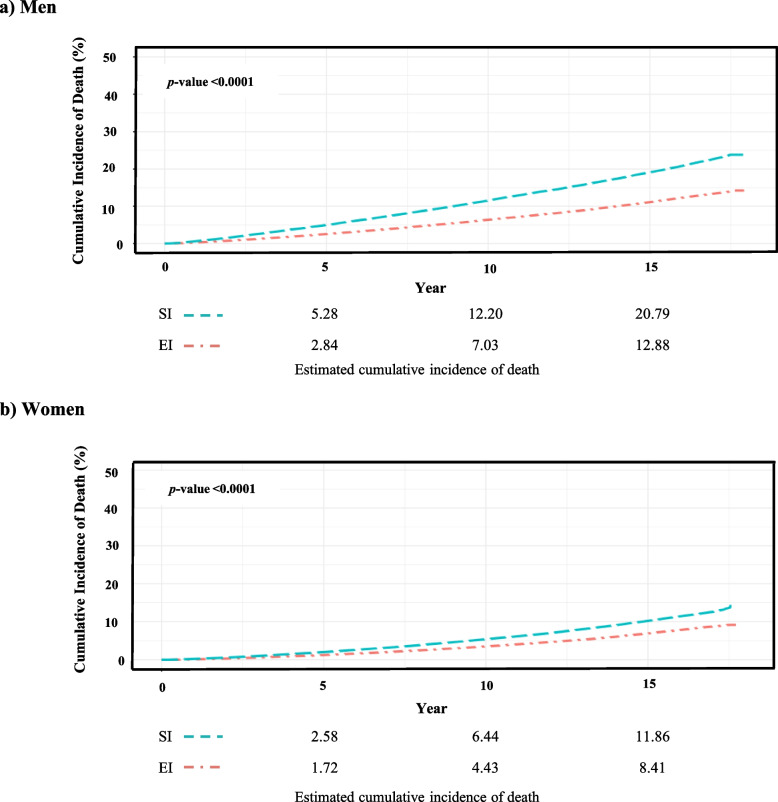
Cumulative incidence of overall death according to insurance type. Legend: SI, self-employed insured group; EI, employee insured group

This study included a follow-up period of an average of 16.6 years for a total of 452,706 individuals. During the follow-up period, 59,816 (13.2%) died from various causes. Cancer was the most frequent cause of death, followed by CVD and CbVD. Appendix 1 provides a detailed breakdown of the causes of death (cancer: 37.1%, CVD: 9.6%, and CbVD: 9.1%). A greater proportion of SI individuals died during follow-up than EI individuals (21.9% of SI and 13.2% of EI for men; 12.1% of SI and 8.4% of EI for women; *p* < 0.0001; Fig. 2). After adjusting for age, smoking status, drinking status, physical activity status, BMI, systolic blood pressure, fasting glucose, total cholesterol, ALT, residential area, and CCI, HRs (95% CIs) for the overall death of SI group were 1.13 (1.10–1.15) for men and 1.18 (1.15–1.22) for women, compared to EI group of the same sex (Table 2). Additionally, statistically significant increases were identified in cause-specific death risk for the SI group compared to the EI group, for causes including cancer, CbVD, intentional self-harm, and external causes for both sexes (Table 3).

**Table 2:** Cox proportional hazard regression analysis for overall death according to insurance type (2002–2019)

HR (95% CI)	Employee insured	Self-employed insured
**Men**		
Model 1	1	1.20 (1.17—1.22)
Model 2	1	1.12 (1.10—1.15)
Model 3	1	1.13 (1.10—1.15)
**Women**		
Model 1	1	1..22 (1.18—1.25)
Model 2	1	1.18 (1.15—1.22)
Model 3	1	1.18 (1.15—1.22)

Model 1: adjusted for age

Model 2: adjusted for smoking status (never, former, and current), alcohol consumption (rare, moderate, and heavy), physical activity (rare, sometimes, and regular), residential area (Seoul capital, other metropolitan, and non-metropolitan), added to Model 1

Model 3: adjusted for systolic blood pressure, body mass index, fasting glucose, alanine aminotransferase, total cholesterol, and Charlson’s comorbidity index (0, 1, 2, ≥ 3), added to Model 2

*HR *Hazard ratio, *CI *Confidence interval

**Table 3:** Cox proportional hazard regression analysis for overall and cause-specific death according to insurance type (Model 3; 2002–2019)

	**HR (95% CI)**	
**Cause of Death**	**Employee-insured**	**Self-employed insured**
**Men**		
Cancer	1	1.06 (1.02–1.09)
Cardiovascular disease	1	1.03 (0.96–1.11)
Cerebrovascular disease	1	1.21 (1.13–1.30)
Pneumonia	1	1.12 (1.02–1.24)
Intentional self-harm	1	1.14 (1.03–1.26)
External cause	1	1.27 (1.17–1.37)
**Women**		
Cancer	1	1.12 (1.06–1.17)
Cardiovascular disease	1	1.20 (1.10–1.30)
Cerebrovascular disease	1	1.21 (1.12–1.32)
Pneumonia	1	1.10 (0.97–1.26)
Intentional self-harm	1	1.35 (1.14–1.59)
External cause	1	1.21 (1.07–1.37)

Adjusted for age, smoking status (never, former, and current), alcohol consumption (rare, moderate, and heavy), physical activity (rare, sometimes, and regular), residential area (Seoul capital, other metropolitan, and non-metropolitan), systolic blood pressure, body mass index, fasting glucose, alanine aminotransferase, total cholesterol, and Charlson’s comorbidity index (0, 1, 2, ≥ 3)

*CI *Confidence interval, *HR *Hazard ratio

### Overall and cause-specific mortality by economic status

**Fig. 3:**
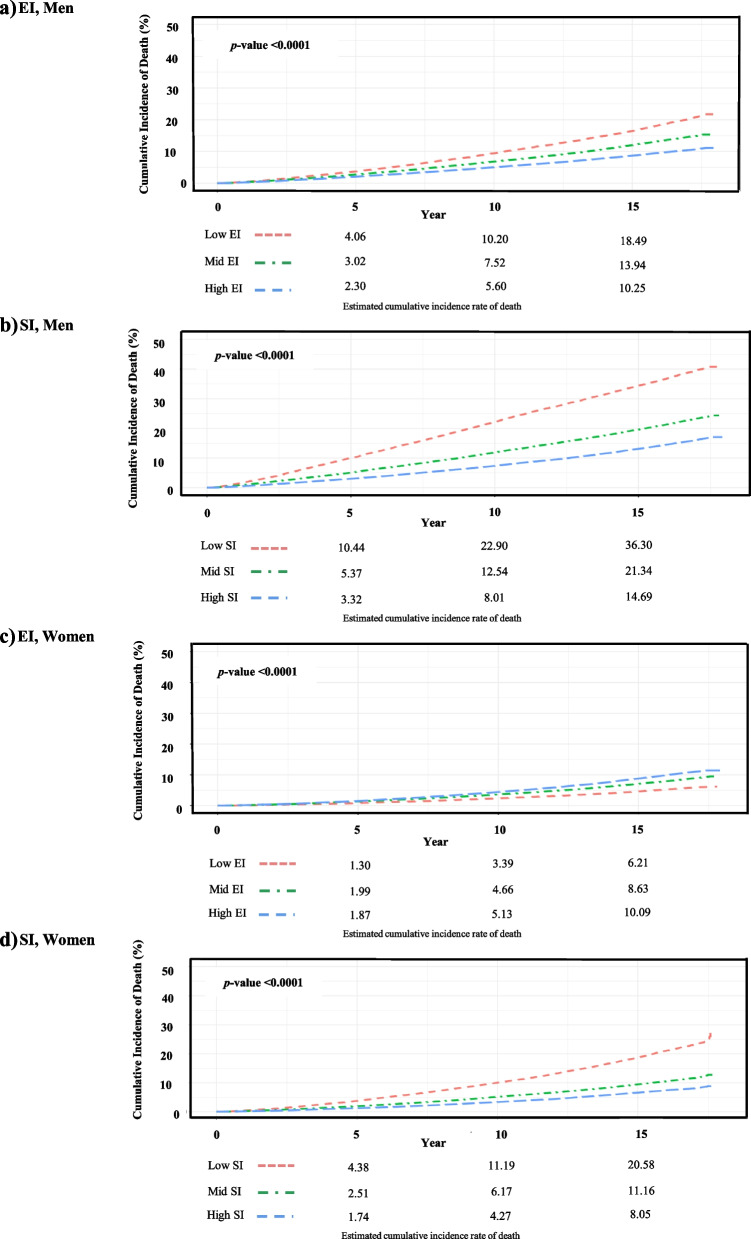
Cumulative incidence of overall death according to insurance type and economic status. Legend: SI, self-employed insured group; EI, employee insured group

Overall and cause-specific mortality rates were also analyzed according to economic status (low, mid, and high) categorized by insurance premiums within each insurance type (EI and SI; Fig. 3 and Table 4). In Fig. 3, individuals with lower economic status had a higher cumulative incidence of overall mortality compared to those with higher economic status, except for the women in the EI group. Among women in the EI group, the high-EI group had the highest and the low-EI group had the lowest cumulative incidence of overall death, with statistically significant differences between all three groups (high-EI group: 10.6% vs. mid-EI group: 8.5% vs. low-EI group: 5.7%,* p* < 0.0001).

**Table 4:** Cox proportional hazard regression analysis for overall and cause-specific death according to economic status (Model 3; 2002–2019)

**a) Employee insured group**				
	**HR (95% CI)**			
**Cause of Death**	**High EI**	**Mid EI**	**Low EI**	**p-trend**
**Men**				
Overall	1	1.25 (1.21—1.29)	1.37 (1.33—1.42)	< 0.001
Cancer	1	1.23 (1.17—1.29)	1.34 (1.28—1.41)	< 0.001
Cardiovascular disease	1	1.15 (1.04—1.27)	1.25 (1.12—1.39)	< 0.001
Cerebrovascular disease	1	1.25 (1.12—1.40)	1.30 (1.15—1.47)	< 0.001
Pneumonia	1	1.11 (0.95—1.30)	1.25 (1.06—1.48)	< 0.001
Intentional self-harm	1	1.62 (1.41—1.86)	1.87 (1.61—2.17)	< 0.001
External cause	1	1.47 (1.30—1.65)	1.64 (1.44—1.86)	< 0.001
**Women**				
Overall	1	1.06 (1.01—1.11)	1.07 (1.02—1.13)	< 0.001
Cancer	1	1.02 (0.94—1.11)	1.03 (0.95—1.13)	< 0.001
Cardiovascular disease	1	1.17 (1.01—1.35)	1.20 (1.01—1.41)	< 0.001
Cerebrovascular disease	1	1.07 (0.93—1.24)	1.16 (0.99—1.36)	< 0.001
Pneumonia	1	0.97 (0.77—1.21)	0.98 (0.75—1.28)	< 0.001
Intentional self-harm	1	1.06 (0.80—1.40)	0.71 (0.52—0.98)	0.003
External cause	1	1.06 (0.85—1.31)	1.24 (0.98—1.56)	0.006
**b) Self-employed insured group**				
	**HR (95% CI)**			
**Cause of Death**	**High SI**	**Mid SI**	**Low SI**	**p-trend**
**Men**				
Overall	1	1.33 (1.28—1.38)	1.56 (1.59—1.63)	< 0.001
Cancer	1	1.21 (1.14—1.28)	1.39 (1.21—1.39)	< 0.001
Cardiovascular disease	1	1.30 (1.14—1.48)	1.60 (1.38—1.84)	< 0.001
Cerebrovascular disease	1	1.58 (1.38—1.80)	1.62 (1.40—1.87)	< 0.001
Pneumonia	1	1.21 (1.01—1.45)	1.41 (1.17—1.71)	< 0.001
Intentional self-harm	1	1.55 (1.29—1.86)	1.84 (1.49—2.27)	< 0.001
External cause	1	1.45 (1.26—1.67)	2.03 (1.73—2.38)	< 0.001
**Women**				
Overall	1	1.18 (1.12—1.24)	1.25 (1.19—1.31)	< 0.001
Cancer	1	1.03 (0.94—1.12)	1.11 (1.02—1.22)	< 0.001
Cardiovascular disease	1	1.30 (1.12—1.52)	1.45 (1.25—1.68)	< 0.001
Cerebrovascular disease	1	1.28 (1.09—1.45)	1.28 (1.11—1.48)	< 0.001
Pneumonia	1	1.04 (0.81—1.33)	1.32 (1.05—1.66)	< 0.001
Intentional self-harm	1	1.22 (0.92—1.62)	1.69 (1.26—2.28)	< 0.001
External cause	1	1.25 (1.01—1.55)	1.19 (0.95—1.50)	< 0.001

Adjusted for age, smoking status (never, former, and current), alcohol consumption (rare, moderate, and heavy), physical activity (rare, sometimes, and regular), residential area (Seoul capital, other metropolitan, and non-metropolitan), systolic blood pressure, body mass index, fasting glucose, alanine aminotransferase, total cholesterol, and Charlson’s comorbidity index (0, 1, 2, ≥ 3)

*CI *Confidence interval, *HR *Hazard ratio, *EI* Employee insured, *SI *Self-employed insured

P-trend from Cochrane-Armitage test for trend of group of insurance premium and risk of cause-specific death

However, after adjusting for age, smoking status, drinking status, physical activity status, BMI, systolic blood pressure, fasting glucose, total cholesterol, ALT, residential area, and CCI, individuals in lower economic status had a significantly higher risk of overall mortality compared to those in the high economic status, regardless of sex and insurance type (adjusted HR [95% CI] of men in the mid- and low-EI groups, 1.25 [1.21–1.29] and 1.37 [1.33–1.42], respectively; women in the mid- and low-EI groups, 1.06 [1.01–1.11] and 1.07 [1.02–1.13], respectively; men in the mid- and low-SI groups, 1.33 [1.28–1.38] and 1.56 [1.59–1.63], respectively; women in the mid- and low-SI groups, 1.18 [1.12–1.31] and 1.25 [1.19–1.31], respectively).

Analyses of the specific causes of death showed different results between men and women. Men with lower economic status showed significantly higher risk of death from cancer, CVD, CbVD, pneumonia, intentional self-harm, and external causes regardless of health insurance type. For women, the pattern differed. Only mortality from CVD showed a significant increase across both EI and SI groups with lower economic status (adjusted HRs [95% CI] of women in the low-EI group, 1.20 [1.01–1.41] and women in the low-SI group, 1.45 [1.25–1.68]). Women in the low-SI group specifically exhibited an increased risk of death from CbVD, pneumonia, and intentional self-harm (adjusted HR [95% CI] of women in the low-SI group, 1.28 [1.11–1.48], 1.32 [1.05–1.66], and 1.69 [1.26–2.28]). However, women in the low-EI group showed significantly decreased risk of death from intentional self-harm compared to those with higher economic status (adjusted HR [95% CI] of women in the low-EI group, 0.71 [0.52–0.98]; Table 4).

Subgroup analyses categorized the individuals by prior diagnoses of cancer, CVD, and CbVD to see if it affected their death risk (Appendix 2). Individuals with a history of cancer had elevated cancer mortality risk only among men with lower economic status, whereas those with a history of CVD had a significantly higher CVD death risk only in the low-SI groups regardless of sex. These findings, despite variations across causes of death and subgroups, support the main conclusion that individuals with lower economic status had an increased risk of overall mortality and cause-specific mortality compared to those with higher economic status, regardless of health insurance type.

## Discussion

This large nationwide cohort study of 452,706 individuals found significantly higher overall and cause-specific mortality rates among those enrolled in the SI group than among those in the EI group. Even after adjusting for various demographic, lifestyle, and clinical factors, individuals in the SI group had a 13–18% higher risk of death than those in the EI group. Additionally, those with lower economic status, proxied by premium levels, had an increased risk of overall and cause-specific mortality in a dose–response manner, regardless of insurance type. This finding aligns with the initial hypothesis of the study.

Similar to our findings, some studies identify vulnerability among populations with limited health insurance coverage or lower SES [23–25]. A Swedish study found 16% lower CVD mortality and 26% lower suicide mortality for self-employed versus paid employees [23]. A systematic review and meta-analysis of 42 studies showed that unemployment is also strongly linked to increased mortality, with a 63% higher risk of death among unemployed adults compared with employed adults [24]. A US study using National Health Interview Survey data revealed a 17% lower mortality risk for those with private insurance but a 21% higher risk for those with public insurance versus the uninsured [25]. These mixed results highlight the complex interplay between employment, insurance factors, and health outcomes across SES contexts.

There are several possible explanations for the observed higher overall and cause-specific mortality among individuals in the SI group and those with a lower economic status in our Korean cohort, in view of the social determinants of health framework. First, in line with prior studies demonstrating the combined effect of multiple unhealthy lifestyles and SES on mortality [26], the observed higher prevalence of mortality risk factors in the SI group, such as current smoking and heavy alcohol consumption, and the higher burden of chronic diseases, such as cancer, CVD, and CbVD, could have contributed to the elevated mortality risk in the SI group. Second, disparities in healthcare access based on economic status, potentially reflected by health insurance type and premium, may play a role as an unmeasured confounding factor. Previous Korean studies have shown that the SI group has lower outpatient medical utilization and higher unmet medical needs compared to the EI group [7]. Conversely, previous studies have shown that having private insurance in addition to the NHIS is associated with increased outpatient costs, inpatient utilization, and lower mortality, particularly among high-income individuals who are more likely to have private insurance [27, 28]. According to these results, since Korea’s NHI covers only 64.5% of the total medical expenses (as of 2021), those with private insurance or high incomes may have access to expensive treatments, procedures, and preventive screenings not covered by public insurance, potentially lowering their mortality risk [29].

Our study also yielded unexpected findings. Women with lower EI displayed a decreased risk of intentional self-harm mortality compared to those with higher EI, which is contrary to existing literature linking low income and financial hardship with suicide risk [30]. Moreover, individuals with a history of CVD in the low-SI group had a significantly higher risk of pneumonia mortality compared to those in the high-SI group. While research on SES and pneumonia-related death is limited, studies have identified low income as a risk factor and determined its relationship with CVD [31–33]. This suggests that specific health conditions and mortality risk might interact with insurance factors to disproportionately impact specific subgroups.

However, some limitations of this study should be considered when interpreting the findings. First, pre-selection bias is inherent to observational studies using existing datasets, including the NHIS-HEALS cohort. While the cohort offers a random selection to eliminate the selection bias, it cannot be eliminated entirely. Second, potential discrepancies exist between the recorded administrative data and the actual diagnoses or causes of death. Studies have estimated only 60% consistency between Korean death certificates and medical records, indicating uncertainty regarding the causes [34]. Third, this study captured insurance status at a single point and was unable to follow potential shifts during study duration. However, research suggests even temporary low-income status can confer long-term mortality risk [35]. Fourth, there was lack of detailed data on mental health, social relationships, occupation, education, and other SES determinants, which are all crucial for a comprehensive understanding and analysis of the risk of suicide and external-cause mortality [36–38]. Lastly, our cohort could not include the Medical Aid beneficiaries, which constitute approximately 2.9% of the population of individuals receiving government subsistence support [39]. This limits the generalizability of the results to the most socially and economically vulnerable subgroups.

Despite these limitations, our study has notably strengths due to its large cohort size, extensive follow-up duration exceeding 16 years, and the ability to compare insurance type and economic proxy groups while adjusting for various confounders. The cause-specific analysis provided valuable insights into disease-based disparities associated with insurance and economic factors. Future research is needed to elucidate the causal relationships and capture unmeasured risk profiles. Nevertheless, this study clearly demonstrates the vulnerability of individuals in the SI group and those with lower SES. These findings highlight the need for targeted policy interventions aimed at specific risk factors and causes of death. This could include providing targeted healthcare services to high-risk populations, such as prevention and screening for those at high risk for intentional self-harm or implementation of programs to prevent pneumonia in low-premium SI enrollees with a history of CVD. Additionally, these findings underscore the importance of incorporating such considerations into public health policy development.

## Conclusion

In conclusion, this nationwide study confirms that individuals enrolled in SI and those with lower economic status faced significantly higher risks of overall and cause-specific mortality compared to those in the EI group. While further research is needed to elucidate the causal mechanisms, these observed disparities necessitate the development of targeted healthcare strategies. These strategies should address risk factors at both the individual, and population levels through public health policy changes to ensure equitable health outcomes across the socioeconomic strata.

### Supplementary Information

Supplementary Material 1: Appendix 1. Number and percentage of deaths by cause during the study duration (2002~2003). Table of number and percentage of all deaths by cause during the study duration.

Supplementary Material 2: Appendix 2. Cox proportional hazard regression analysis for overall and cause-specific death according to economic status and insurance type by subgroup (2002~2019). Results of Cox proportional hazard regression analysis for cause-specific death according to economic status and insurance type according to history of cancer, CVD, and CbVD.

## Data Availability

The data that support the findings of this study are available from [National Health Insurance Service] but restrictions apply to the availability of these data, which were used under license for the current study, and so are not publicly available. Data are however available from the authors upon reasonable request and with permission of [Hee-Taik,Kang].

## Abbreviations

NHI: National Health Insurance
EI: Employee-insured
SI: Self-employed-insured
HR: Hazard ratio
SES: Socioeconomic status
NHIS-HEALS: National Health Insurance Service-Health Screening
NHSP: National health screening program
NHIS: National Health Insurance Service
ICD: International Classification of Diseases
CVD: Cardiovascular disease
CbVD: Cerebrovascular disease
BMI: Body mass index
CCI: Charlson comorbidity index
ALT: Alanine aminotransferase
