# Supplementary material for: Disparities in cause-specific mortality by health insurance type and premium: evidence from Korean NHIS-HEALS cohort study, 2002–2019
Source: BMC Public Health. 2024 Jun 12;24:1577. doi: 10.1186/s12889-024-19088-3 (PMC11167746; doi:10.1186/s12889-024-19088-3)
Supplement: Supplementary file 1 — Supplementary Material 1: Appendix 1. Number and percentage of deaths by cause during the study duration (2002~2003). Table of number and percentage of all deaths by cause during the study duration. [file 12889_2024_19088_MOESM1_ESM.docx]

**Appendix 1.** Number and percentage of deaths by cause during the study duration (2002–2019)

| Cause of death (%) | Men | Women | Total |
| --- | --- | --- | --- |
| Overall | 39,069 | 20,747 | 59,816 |
| Cancer | 15,839 (40.5) | 6369 (30.7) | 22,208 (37.1) |
| Cardiovascular disease | 3420 (8.8) | 2293 (11.1) | 5713 (9.6) |
| Cerebrovascular disease | 3075 (7.9) | 2364 (11.4) | 5439 (9.1) |
| Pneumonia | 1673 (4.3) | 929 (4.5) | 2602 (4.4) |
| Intentional self-harm | 1797 (4.6) | 574 (2.8) | 2371 (4.0) |
| External Cause | 2688 (6.9) | 1025 (4.9) | 3713 (6.2) |
| Others | 10,577 (29.3) | 7193 (34.7) | 17,770 (29.7) |
