# Supplementary material for: Disparities in cause-specific mortality by health insurance type and premium: evidence from Korean NHIS-HEALS cohort study, 2002–2019
Source: BMC Public Health. 2024 Jun 12;24:1577. doi: 10.1186/s12889-024-19088-3 (PMC11167746; doi:10.1186/s12889-024-19088-3)
Supplement: Supplementary file 2 — Supplementary Material 2: Appendix 2. Cox proportional hazard regression analysis for overall and cause-specific death according to economic status and insurance type by subgroup (2002~2019). Results of Cox proportional hazard regression analysis for cause-specific death according to economic status and insurance type according to history of cancer, CVD, and CbVD. [file 12889_2024_19088_MOESM2_ESM.docx]

**Appendix 2.** Cox proportional hazard regression analysis for overall and cause-specific death according to economic status and insurance type by subgroup (Model 3; 2002–2019)

1. Group with cancer history

|  | **HR (95% CI)** | | |
| --- | --- | --- | --- |
| **Cause of Death** | **High EI** | **Mid EI** | **Low EI** |
| **Men** |  |  |  |
| Overall | 1 | 1.21 (1.09—1.35) | 1.35 (1.21—1.52) |
| Cancer | 1 | 1.27 (1.10—1.45) | 1.37 (1.17—1.60) |
| Cardiovascular disease | 1 | 1.53 (1.04—1.27) | 1.81 (1.15—2.87) |
| Cerebrovascular disease | 1 | 0.69 (0.41—1.14) | 1.40 (0.87—2.25) |
| Pneumonia | 1 | 1.26 (0.75—2.14) | 1.20 (0.61—2.35) |
| Intentional self-harm | 1 | 2.09 (1.15—3.81) | 1.67 (0.81—3.44) |
| External cause | 1 | 1.71 (1.00—2.90) | 1.54 (0.81—2.94) |
| **Women** |  |  |  |
| Overall | 1 | 1.12 (0.95—1.34) | 1.21 (0.99—1.47) |
| Cancer | 1 | 1.02 (0.80—1.30) | 1.21 (0.93—1.57) |
| Cardiovascular disease | 1 | 1.58 (0.82—3.02) | 1.54 (0.70—3.38) |
| Cerebrovascular disease | 1 | 1.34 (0.72—2.52) | 1.50 (0.74—3.01) |
| Pneumonia | 1 | 2.30 (1.02—5.22) | 1.42 (0.49—4.13) |
| Intentional self-harm | 1 | 0.87 (0.25—3.01) | 0.47 (0.09—2.31) |
| External cause | 1 | 1.01 (0.40—2.56) | 0.34 (0.08—1.56) |

|  | **HR (95% CI)** | | |
| --- | --- | --- | --- |
| **Cause of Death** | **High SI** | **Mid SI** | **Low SI** |
| **Men** |  |  |  |
| Overall | 1 | 1.37 (1.22—1.50) | 1.34 (1.17—1.54) |
| Cancer | 1 | 1.36 (1.16—1.60) | 1.25 (1.04—1.51) |
| Cardiovascular disease | 1 | 1.27 (0.77—2.08) | 1.27 (0.72—2.22) |
| Cerebrovascular disease | 1 | 0.69 (1.02—2.95) | 1.44 (0.79—2.63) |
| Pneumonia | 1 | 1.06 (0.58—1.93) | 1.34 (0.71—2.53) |
| Intentional self-harm | 1 | 1.77 (0.86—3.66) | 2.19 (0.98—4.91) |
| External cause | 1 | 1.54 (0.83—2.84) | 1.07 (0.48—2.40) |
| **Women** |  |  |  |
| Overall | 1 | 1.32 (1.12—1.57) | 1.15 (0.95—1.37) |
| Cancer | 1 | 1.09 (0.85—1.38) | 1.07 (0.82—1.39) |
| Cardiovascular disease | 1 | 2.78 (1.30—5.93) | 2.58 (1.17—5.68) |
| Cerebrovascular disease | 1 | 1.47 (0.83—2.60) | 1.09 (0.59—2.02) |
| Pneumonia | 1 | 1.08 (0.45—2.56) | 0.88 (0.36—2.19) |
| Intentional self-harm | 1 | 2.35 (0.67—8.24) | 1.85 (0.48—7.08) |
| External cause | 1 | 2.63 (0.89—7.79) | 1.51 (0.44—5.25) |

1. Group with cardiovascular disease history

|  | **HR (95% CI)** | | |
| --- | --- | --- | --- |
| **Cause of Death** | **High EI** | **Mid EI** | **Low EI** |
| **Men** |  |  |  |
| Overall | 1 | 1.08 (0.96—1.22) | 1.30 (1.15—1.48) |
| Cancer | 1 | 1.10 (0.90—1.33) | 1.31 (1.06—1.63) |
| Cardiovascular disease | 1 | 0.99 (0.73—1.34) | 1.18 (0.85—1.65) |
| Cerebrovascular disease | 1 | 1.41 (0.97—2.06) | 1.66 (1.11—2.49) |
| Pneumonia | 1 | 1.50 (0.83—2.73) | 1.25 (0.59—2.68) |
| Intentional self-harm | 1 | 1.51 (0.76—2.97) | 2.42 (1.22—4.80) |
| External cause | 1 | 1.09 (0.65—1.80) | 1.25 (0.71—2.22) |
| **Women** |  |  |  |
| Overall | 1 | 1.02 (0.88—1.18) | 1.35 (1.13—1.61) |
| Cancer | 1 | 1.23 (0.88—1.72) | 1.89 (1.30—2.72) |
| Cardiovascular disease | 1 | 0.79 (0.54—1.16) | 1.21 (0.77—1.88) |
| Cerebrovascular disease | 1 | 1.05 (0.70—1.56) | 1.37 (0.84—2.24) |
| Pneumonia | 1 | 1.05 (0.50—2.20) | 1.85 (0.81—4.19) |
| Intentional self-harm | 1 | 1.97 (0.78—5.01) | 0.83 (0.21—3.26) |
| External cause | 1 | 0.64 (0.28—1.46) | 1.95 (0.94—4.05) |
|  | **HR (95% CI)** | | |
| **Cause of Death** | **High SI** | **Mid SI** | **Low SI** |
| **Men** |  |  |  |
| Overall | 1 | 1.39 (1.21—1.58) | 1.54 (1.33—1.78) |
| Cancer | 1 | 1.05 (0.83—1.33) | 1.02 (0.78—1.34) |
| Cardiovascular disease | 1 | 1.57 (1.13—2.18) | 1.47 (1.01—2.14) |
| Cerebrovascular disease | 1 | 2.59 (1.69—3.97) | 1.95 (1.19—3.18) |
| Pneumonia | 1 | 1.76 (0.95—3.26) | 2.45 (1.29—4.64) |
| Intentional self-harm | 1 | 1.87 (0.89—3.92) | 2.38 (1.05—5.35) |
| External cause | 1 | 1.03 (0.53—2.04) | 1.55 (0.76—3.19) |
| **Women** |  |  |  |
| Overall | 1 | 1.22 (1.04—1.42) | 1.22 (1.05—1.42) |
| Cancer | 1 | 1.17 (0.86—1.60) | 1.20 (0.87—1.65) |
| Cardiovascular disease | 1 | 1.49 (1.00—2.21) | 1.59 (1.08—2.35) |
| Cerebrovascular disease | 1 | 1.08 (0.70—1.64) | 1.01 (0.66—1.53) |
| Pneumonia | 1 | 1.46 (0.56—3.81) | 2.42 (1.01—5.81) |
| Intentional self-harm | 1 | 1.53 (0.54—4.37) | 1.06 (0.34—3.35) |
| External cause | 1 | 1.41 (0.66—3.01) | 1.14 (0.51—2.56) |

1. Group with cerebrovascular disease history

|  | **HR (95% CI)** | | |
| --- | --- | --- | --- |
| **Cause of Death** | **High EI** | **Mid EI** | **Low EI** |
| **Men** |  |  |  |
| Overall | 1 | 1.09 (0.94—1.26) | 1.40 (1.19—1.64) |
| Cancer | 1 | 0.98 (0.74—1.29) | 1.43 (1.08—1.91) |
| Cardiovascular disease | 1 | 1.12 (0.72—1.75) | 1.42 (0.88—2.30) |
| Cerebrovascular disease | 1 | 1.32 (0.95—1.83) | 1.62 (1.13—2.31) |
| Pneumonia | 1 | 1.65 (0.87—3.13) | 1.28 (0.58—2.83) |
| Intentional self-harm | 1 | 0.75 (0.30—1.87) | 1.31 (0.56—3.08) |
| External cause | 1 | 0.87 (0.44—1.72) | 0.90 (0.42—1.96) |
| **Women** |  |  |  |
| Overall | 1 | 1.14 (0.96—1.36) | 1.17 (0.92—1.47) |
| Cancer | 1 | 1.03 (0.69—1.53) | 1.00 (0.60—1.67) |
| Cardiovascular disease | 1 | 1.19 (0.70—2.00) | 1.64 (0.86—3.16) |
| Cerebrovascular disease | 1 | 1.44 (0.99—2.09) | 1.30 (0.79—2.15) |
| Pneumonia | 1 | 1.27 (0.60—2.67) | 1.00 (0.33—3.07) |
| Intentional self-harm | 1 | 1.08 (0.24—5.01) | 3.45 (0.84—14.18) |
| External cause | 1 | 1.13 (0.44—2.92) | 1.61 (0.54—4.80) |

|  | **HR (95% CI)** | | |
| --- | --- | --- | --- |
| **Cause of Death** | **High SI** | **Mid SI** | **Low SI** |
| **Men** |  |  |  |
| Overall | 1 | 1.25 (1.07—1.45) | 1.33 (1.13—1.57) |
| Cancer | 1 | 1.11 (0.82—1.51) | 1.16 (0.83—1.63) |
| Cardiovascular disease | 1 | 0.87 (0.51—1.48) | 1.27 (0.75—2.15) |
| Cerebrovascular disease | 1 | 1.48 (1.08—2.03) | 1.24 (0.87—1.77) |
| Pneumonia | 1 | 1.31 (0.66—2.63) | 1.03 (0.47—2.26) |
| Intentional self-harm | 1 | 2.22 (0.98—5.35) | 1.44 (0.49—4.26) |
| External cause | 1 | 2.04 (1.02—4.10) | 2.17 (0.99—4.75) |
| **Women** |  |  |  |
| Overall | 1 | 1.21 (0.99—1.47) | 1.29 (1.07—1.57) |
| Cancer | 1 | 0.97 (0.63—1.50) | 1.01 (0.65—1.56) |
| Cardiovascular disease | 1 | 0.87 (0.49—1.53) | 1.18 (0.71—1.97) |
| Cerebrovascular disease | 1 | 1.40 (0.92—2.13) | 1.56 (1.04—2.34) |
| Pneumonia | 1 | 1.03 (0.40—2.67) | 1.14 (0.46—2.81) |
| Intentional self-harm | 1 | 2.95 (0.30—28.8) | 4.20 (0.44—40.3) |
| External cause | 1 | 0.82 (0.27—2.49) | 0.75 (0.25—2.31) |

Note: Adjusted for age, smoking status (never, former, and current), alcohol consumption (rare, moderate, and heavy), physical activity (rare, sometimes, and regular), residential area (Seoul capital, other metropolitan, and non-metropolitan), systolic blood pressure, body mass index, fasting glucose, alanine aminotransferase, total cholesterol, and Charlson’s comorbidity index (0, 1, 2, ≥3); CI, confidence interval; HR, hazard ratio; EI, employee insured; SI, self-employed insured.
